# An ABC Transporter Is Involved in the Silicon-Induced Formation of Casparian Bands in the Exodermis of Rice

**DOI:** 10.3389/fpls.2017.00671

**Published:** 2017-04-28

**Authors:** Martin Hinrichs, Alexander T. Fleck, Eline Biedermann, Ngoc S. Ngo, Lukas Schreiber, Manfred K. Schenk

**Affiliations:** ^1^Institute of Plant Nutrition, Faculty of Natural Science, Leibniz Universität HannoverHannover, Germany; ^2^Institute of Cellular and Molecular Botany, Department of Ecophysiology, University of BonnBonn, Germany

**Keywords:** radial oxygen loss, Fe uptake, phenylpropanoid metabolism, CaMV 35s enhancer trap lines, LRR, bypass flow

## Abstract

Silicon (Si) promotes the formation of Casparian bands (CB) in rice and reduces radial oxygen loss (ROL). Further transcriptomic approaches revealed several candidate genes involved in the Si-induced formation of CB such as ATP binding cassette (ABC) transporter, Class III peroxidases, ligases and transferases. Investigation of these genes by means of overexpression (OE) and knockout (KO) mutants revealed the contribution of the ABC transporter (*OsABCG25*) to CB formation in the exodermis, which was also reflected in the expression of other *OsABCG25* in the Si-promoted formation of CB genes related to the phenylpropanoid pathway, such as phenylalanine-ammonia-lyase, diacylglycerol O-acyltransferase and 4-coumarate-CoA ligase. Differential CB development in mutants and Si supply also affected the barrier function of the exodermis. OE of the ABC transporter and Si supply reduced the ROL from roots and Fe uptake. No effect on ROL and Fe uptake could be observed for the KO mutant. The presented research confirms the impact of the *OsABCG25* in the Si-promoted formation of CB and its barrier functions.

## Introduction

Silicon (Si) is not an essential element, but has several beneficial effects on plant growth. It is one of the most abundant elements in the soil surface, with a soil solution concentration of 2.5–20 mg Si^∗^L^-1^ silicic acid (Bogdan and Schenk, 2008; Marxen et al., 2016). Plants differ in their ability to accumulate Si and rice is known to be a strong accumulator, containing Si in even higher concentrations than nitrogen, potassium or calcium (Epstein, 1994). One of the Si effects is the formation of mechanical barriers in leaves and roots (Cai et al., 2009). Rice is cultivated under flooded (anaerobic) and unflooded (aerobic) conditions. Under submerged conditions adventitious rice roots develop aerenchyma by the lysis of cell walls in the cortex to ensure oxygen supply to the root tips from the shoot (Nishiuchi et al., 2012). However, oxygen diffuses from the aerenchyma into the rhizosphere which is hampered by the Casparian band (CB) in the exodermis. The CB development starts about 5–8 cm behind the root tip in the anticlinal cell walls and is mostly completed at a 12–13 cm distance from the root tip, reducing the radial oxygen loss (ROL) from roots (Steudle, 2000; Vaculík et al., 2009; Fleck et al., 2011). The CBs in the exodermis are also thought to reduce the inflow of freely available ions from the soil solution into the cortex (Yeo et al., 1999; Gong et al., 2006; Faiyue et al., 2010). The CB development in the exodermis is stimulated by the Si supply in rice and also in other species, such as *Zea mais, Allium cepa, Tradescantia virginiana*, and *Guizotia abyssinica*, restricting the ROL to the first 5 cm behind the root tip in rice (Fleck et al., 2015). Furthermore, it was shown that Si supply reduces the Fe concentration in shoot matter (Ma and Takahashi, 1990; Dufey et al., 2013). It is hypothesized that Si-stimulated CB formation reduces the Fe flow into the cortex, where it is bound to Deoxymugineic acid and taken up via yellow stripe- like transporters (Yehuda et al., 1996; Nozoye et al., 2011).

The CBs occur in the endodermis of all species and in the exodermis of most species, including rice, maize and onion, but not in *A. thaliana* (Schreiber et al., 1999; Ma and Peterson, 2003; Naseer et al., 2012; Fleck et al., 2015). The main components are lignin and suberin (Schreiber et al., 2005b; Naseer et al., 2012). Suberin is a biopolymer consisting of aliphatic components (ω-hydroxy acids, α, ω-dicarboxylic acids, fatty acids, alcohols) and aromatic components (ferulic acid; Franke et al., 2005). The CB formation in the endodermis of *A. thaliana* starts with the deposition of lignin hampering the flux from the cortex into the stele. In the second step, suberin is deposited (Naseer et al., 2012). The CB in the exodermis of rice is formed by simultaneous incorporation of lignin and suberin into anticlinal cell walls (Kotula et al., 2009; Fleck et al., 2015). These CB compounds are provided by the phenylpropanoid pathway and it has been shown that some of the genes, like phenylalanine-ammonia-lyase (PAL), 4-coumarate-CoA ligase (4CL), glycerol-3-phosphate acyltransferase (AT), diacylglycerol O-acyltransferase (DGOAT), ATP binding cassette (ABC) transporter and class III peroxidases (POD) involved in this secondary metabolism pathway are upregulated through Si in roots (Fleck et al., 2011). Additionally, expression of a leucine-rich repeat (LRR) family protein and an ABC transporter (*OsABCG25*; LOC_Os10g30610) was enhanced. The transporter *OsABCG25* was suggested to be involved in the transport of monolignols or suberin monomers in the Si-induced development of CB in the exodermis. The PAL desaminates phenylalanine to cinnamic acid, which is metabolized via several steps to precursors of lignin and suberin (Zhong et al., 1998; Eckardt, 2002). The 4CL catalyzes the formation of monolignols from coumaroyl-CoA, feruloyl-CoA, or sinapoyl-CoA. Suberin consists of aliphatic and aromatic components where the aliphatic components are provided from fatty acids by POD. The aliphatic and aromatic components, are bound to glycerol by AT, such as DGOAT to suberin monomers. Both monolignols and suberin monomers are most probably transported by ABC transporters into the apoplast (Landgraf et al., 2014; Shiono et al., 2014). The function of these genes in Si-stimulated CB development was studied in overexpression (OE) and knockout (KO) mutants. We observed the involvement of the *OsABCG25* in CB formation and, additionally, investigated the expression of genes related to lignin and suberin metabolism. Furthermore, the barrier function of exodermal CB with regarding ROL from roots and Fe uptake were investigated in these mutants.

## Materials and Methods

### Selection of Rice Mutant Lines

We selected 24 rice mutant lines carrying a T-DNA insertion that contains multimerized cauliflower mosaic virus (CaMV) 35S enhancers leading to an OE of nearby genes (Jeong et al., 2002; Chern et al., 2007). As selection criteria, the T-DNA insertion of the mutant lines should be located within 10,000 bp upstream or downstream of one of eight candidate gene for suberin or lignin synthesis and must not interrupt the sequence of a non-target gene. In contrast to the other lines, the line *1B-14436* carried an insertion in the exon sequence of a candidate gene, resulting in an interrupted transcription of the gene *OsABCG25* (LOC_Os10g30610; Genebank ID: ABB47708.1; Uniprot ID: B9G5Y5). The positions of the T-DNA relative to the target gene were calculated using the GenomeBrowser of the OryGenesDB database^1^ (Droc et al., 2006). The mutant lines selected were ordered from the Pohang University of Science and Technology (Postech; Pohang, Republic of Korea; Jeon et al., 2000) and from the Taiwan Rice Insertional Mutants Database (TRIM; Taiwan). **Table 1** summarizes the mutant lines, the target gene identifiers, the position of the insert relative to the start codon of the target gene, and the supplier of the seeds. Out of these 24 rice mutant lines a total of ten homozygous lines were obtained.

**Table 1:** Mutant lines, target genes, position of the insert relative to the gene and supplier of the seeds.

Mutant line	Target gene	Position relative to start codon of gene	Supplier
*1B-04415*	LOC_Os01g67540	245 upstream	Postech
*3A-14487*	LOC_Os01g67540	759 upstream	Postech
*2D-41110*	LOC_Os02g41680	5270 downstream	Postech
*M0060856*	LOC_Os02g41680	59 downstream	TRIM
*4A-50856*	LOC_Os05g20100	161 downstream	Postech
*5A-00450*	LOC_Os06g16350	3745 upstream	Postech
*5A-00464*	LOC_Os06g16350	3820 upstream	Postech
*3A-01911*	LOC_Os06g16350	10457 upstream	Postech
*M0038578*	LOC_Os06g22080	3644 downstream	TRIM
*3D-01082*	LOC_Os06g22080	9232 downstream	Postech
*3A-01215*	LOC_Os08g02110	392 downstream	Postech
*3A-02897*	LOC_Os08g02110	420 upstream	Postech
*3A-08589*	LOC_Os08g02110	9509 upstream	Postech
*3A-06124*	LOC_Os10g30610	1466 upstream	Postech
*3A-16329*	LOC_Os10g30610	3131 upstream	Postech
*3A-16331*	LOC_Os10g30610	3874 upstream	Postech
*3A-02127*	LOC_Os10g30610	4537 upstream	Postech
*3A-60593*	LOC_Os10g30610	4139 upstream	Postech
*2D-00893*	LOC_Os10g30610	5277 downstream	Postech
*M0033740*	LOC_Os11g14050	7665 upstream	TRIM
*M0058854*	LOC_Os11g14050	3592 downstream	TRIM
*2A-20141*	LOC_Os11g14050	1810 downstream	Postech
*M0066685*	LOC_Os11g14050	12599 downstream	TRIM
*1B-14436*	LOC_Os10g30610	Exon	Postech

### Plant Material, Growth Conditions and Harvest, T1 Seeds

Rice (*Oryza sativa* L.) seeds of the insertion lines (**Table 1**) were delivered as T1 seeds containing a mixture of wild type (WT), heterozygous and homozygous mutant plants. Seeds were germinated in tap water for several days for seed propagation and the seedlings were then transferred to 10-L pots containing soil from the local campus and grown submerged in a greenhouse with average temperatures around 28°C and a minimum of 220 μmol M^-2^ s^-1^ light intensity until maturity. After a few weeks, the genotype of the plants was determined using DNA from the leaves and only homozygous mutant plants were further cultivated further. Whole plants were individually enwrapped in plastic sleeves at the time of flowering to prevent crosspollination with other plants.

### Genotyping

The DNA extraction was performed using a crude leaf extract from a NaOH-Tris-extraction method for genotyping (Collard et al., 2007). An amount of 200 mg of a leaf were harvested, transferred to a 2.0-ml tube containing one steel ball and 100 μl of 0.5 M NaOH. The leaf was homogenized in a swing mill for 1 min at 30 Hz and then 900 μl of 0.1 M Tris was added. Samples were centrifuged for 3 min at 13000 × *g*, the supernatant was transferred to a fresh 1.5-ml tube and stored at -20°C.

An amount of 1 μl of the DNA extracted was used in 25 μl PCR reaction mix containing 2.5 μl 10x reaction buffer, 3.6 mM MgCl_2_, 0.2 mM dNTPs (Fermentas, St. Leon-Rot, Germany), 0.75 U *Taq-*DNA-polymerase (DNA cloning service, Hamburg, Germany), 0.25 μM forward primer and 0.25 μM reverse primer. Two PCR runs with different primer combinations were used for each insertion line. A primer pair targeting at genomic regions flanking the insert was used (W-primer pair) in the first PCR, and an insert-specific primer targeting at a sequence near the border of the T-DNA was used together with one primer of the first PCR (I-primer pair) in the second PCR. The PCR products were electrophoretically separated on a 1% agarose gel. The WT plants showed a band with the W-primer pair, homozygous mutant plants were identified by a band with the I-primer pair, while heterozygous plants were characterized by bands with both primer pairs. The PCR runs included both negative controls with water instead of DNA and positive controls with DNA from WT plants. The genotyping primers used for each line are summarized in Supplementary Tables S1, S2.

### Growth Conditions and Harvest, T2 Seeds

The T2 seeds of homozygous mutant plants and corresponding WT plants were germinated in tap water for several days and then placed between two layers of filter paper standing in tap water for 7 days. Seedlings were transferred to nutrient solution in 5-L pots containing 0.43 mM NH_4_NO_3_, 0.32 mM NaH_2_PO_4_, 0.51 mM K_2_SO_4_, 1 mM Ca(NO_3_)_2_, 1.6 mM MgSO_4_, 1.82 μM MnSO_4_, 0.03 μM (NH_4_)_6_Mo_7_O_24_, 9 H_3_BO_3_, 0.6 μM ZnSO_4_, 0.15 μM CuSO_4_, and 35.81 μM Fe^EDDHA^. The pH value was adjusted to 6.0 by the addition of 10% H_2_SO_4_ and 1 M KOH and the nutrient solution was renewed weekly for the first 2 weeks. After 2 weeks, the nutrient solution was changed twice a week until harvest. Plants were cultivated in a climate chamber (photoperiod 14/10 h light/dark; temperature, 25/20°C day/night; 75% relative humidity and a light intensity of 220 μmol m^-2^ s^-1^).

The WT seeds and T2 seeds of homozygous mutant plants of the lines *1B-14436* (KO) and *3A-16329* (OE) were germinated and cultivated in nutrient solution as described above, but with two Si treatments, to determine the effect of Si supply. Si was applied as silica gel and Si concentrations were 3 or 30 mg L^-1^ resulting in Si concentrations of 0.1/0.1 and 60/3 mg^∗^g^-1^ shoot / root DM, respectively (Supplementary Figure S1).

The homozygous genotype of the plants was confirmed during the cultivation using DNA from the leaves. After 28 days in nutrient solution, root zones 4–6 cm behind the root tip were harvested and either stored in 70% ethanol at 4°C for subsequent histochemical determination of CB or transferred immediately to liquid nitrogen and stored at -80°C for transcript analysis. Shoot and root were separated, dried at 60°C for 4 days and weighed.

### Histochemical Examination of Roots

Freehand cross sections of adventitious roots fixed in 70% EtOH were stained with 0.1% (w/v) berberine hemisulfate for 60 min, washed three times with distilled water and counterstained with 0.5% (w/v) aniline blue for a further 30 min for detection of CB (Brundrett et al., 1988). Stained sections were mounted in 0.1% (w/v) FeCl_3_ in 50% (v/v) glycerine and examined using an Axioskop fluorescence microscope (Zeiss, Jena, Germany) with UV illumination and excitation filter G 365, chromatic beam splitter FT 395 and barrier filter LP 420. Pictures were taken with the AxioCam MRc (Zeiss) and picture recording software (AxioVision Ac, Version 4.4, Zeiss). Suberin exhibited a blue-white color under UV light. The development of CB in the anticlinal exodermal cell walls was determined and allocated to one of four stages: 0% (stage I), 0–25% (II), 25–50% (III) and 50–100% (IV) development of CB in the anticlinal cell wall of the exodermis.

Five roots without lateral roots were taken from each of the four replicates for cross-sectioning and 20 cells each from five cross sections were used for microscopic examination, therefore, the degree of development of CB was based on 400 cell walls per treatment.

### Transcript Analysis

Frozen root material was ground under liquid nitrogen and total RNA was isolated using TRIsure^®^ Reagent (Bioline, Luckenwalde, Germany), following the manufacturer’s instructions. The RNA quality was determined electrophoretically by 2% non-denaturating agarose gel and fluoretically using a Nanophotometer (Implen, Munich, Germany). The total RNA (1 μg) and random hexamer primers were used to synthesize first-strand cDNA using the Revert Aid^TM^ H Minus Kit (Fermentas, St. Leon-Rot, Germany), following the manufacturer’s instructions for GC-rich templates.

In the qRT-PCR experiments, 100 ng cDNA was used as a template in 25 μl reaction mix containing 2.5 μl 10x buffer, 3.6 mM MgCl_2_, 0.2 mM dNTPs mix (Fermentas, St. Leon-Rot, Germany), 0.25 μl 1:1000 diluted SYBR-Green (Invitrogen, Carlsbad, CA, USA), 0.75 U HotStart-Taq-DNA-Polymerase (DNA cloning service, Hamburg, Germany), and 0.25 μM forward and 0.25 μM reverse primers. The qRT-PCR runs were performed in the CFX96 cycler (Bio-Rad, München, Germany), using an initial 95°C-step for 10 min, followed by 40 cycles of 95°C for 15 s, 60°C for 30 s and 72°C for 30 s, and a final melting curve procedure with a stepwise increment of 1°C ranging from 60 to 95°C.

The eukaryotic elongation factor 1-alpha (eF1-α) was used as an endogenous control due to its stable transcript abundance in rice (Jain, 2009). A list of primer sequences used can be found in Supplementary Table S3. Three technical and three biological replicates were used for each target in qRT-PCR. The relative quantity was calculated using the R-Macro “qpcrmix” (Steibel et al., 2009), based on the 2^-ΔΔCT^ method.

### Cell Wall Isolation and Preparation for Suberin Analysis

Root zones 4–6 cm behind the root tip were harvested and the root surface was scanned using WinRHIZO software (Regent Instruments Inc., Quebec, QC, Canada). The cell wall isolation and preparation was performed as described in detail by Schreiber et al. (1994). Briefly, root zones were washed with H_2_O_dest_ and then incubated at room temperature for 4 days in 1 ml enzyme solution (0.1 M citric acid monohydrate, 1% pectinase (v/v), 1% cellulase (v/v), 0.1% NaN_3_), which was renewed daily. After enzymatic digestion, the non-degradable outer part of the root comprising the exodermal cell wall fraction and the sclerenchyma was separated from the tissue containing the stele by using two forceps under a binocular. The exodermal cell wall material was incubated in enzyme solution for another 2 days to remove any residual cortex material. Subsequently, the isolates were washed with H_2_O_dest_ and incubated in borate buffer (0.01 M sodium borate, pH 9) for 2 days.

Dried isolated cell wall material was extracted for 5 days with a 1:1 mixture of chloroform and methanol, which was changed daily. After the extraction, the isolated samples were dried for 2 h in the desiccator over silica gel. The dry weight was determined just prior to the suberin analysis of isolated samples.

### Suberin Analysis

The dried sample isolates were incubated for 16 h in 1 N methanolic boron trifluoride (MeOH/BF_3_; Fluka/Sigma-Aldrich, St. Louis, MO, USA) at 70°C for transesterification. Saturated NaOH was added to stop the transesterification reaction and to advance the following phase separation. Dotriacontan (C_32_ alkane, 10.025 mg/ 50 ml) was added to each sample as an internal standard. The soluble hydrophobic components were extracted by adding chloroform. The chloroform phase was transferred to a new vial and extraction was repeated three times. The extract was dried with water-free Na_2_SO_4_ and the volume was reduced to 50 μl by evaporation under N_2_ flow.

Samples were derivatized in 20 μl BSTFA (N,N-bis(trimethylsilyl)-trifluoracetamide; Machery-Nagel, Düren, Germany) and 20 μl dry pyridine (GC-grade, Merck, Darmstadt, Germany) for 40 min at 70°C. Pyridine catalyzed the derivatization reaction and BSTFA masked free hydroxyl- and carboxyl-groups forming the corresponding trimethylsilyl derivatives (Schreiber et al., 2005b). Samples were analyzed by gas chromatography (GC; Type: 6890N, Agilent Technologies, Santa Clara, CA, USA) and mass spectroscopy (MS: Type: 5973N, Agilent Technologies, Santa Clara, CA, USA). The GC and MS analyses were performed as described previously in detail in Zeier and Schreiber (1997). The quantification of the monomers was performed using a gas chromatograph combined with a flame ionization detector. Four replicates of each rice line were used.

### Visualization of Radial Oxygen Loss

In order to visualize the ROL, adventitious roots of plants were grown for 28 days in nutrient solution with and without Si supply, as described above. An adventitious root was placed between two acryl glass plates (16 cm × 6 cm; 0.5 cm apart) which were sealed with Plasticine (Pelikan, Hannover, Germany) and the rest of the root system remained in nutrient solution. The space between the plates with the root was filled with 38°C warm semisolid agar medium containing FeS by use of a pipette and the top was sealed with paraffin wax. The medium was prepared by adding 0.8% agar to iron-free nutrient solution and subsequent heating to solubilize the agar. The solution was amended with 1.4 g FeSO_4_ × 7 H_2_O L^-1^ and 0.32 g Na_2_S L^-1^, whereupon a black FeS precipitation developed (Trolldenier, 1988). Finally, the solution was buffered by the addition of 0.5 g CaCO_3_ L^-1^ and adjusted to pH 6.0. The acryl glass plates, held together by clamps, and the plant were fixed using a tripod. The plates were covered with aluminum foil and scans of the plates were taken by a flatbed scanner (Expression 1600, Seiko Epson K.K., Suwa, Nagano, Japan). 12 h after embedding in agar, six roots were investigated for each treatment. The area of ROL was determined using the Fiji imaging software (Schindelin et al., 2012), transforming the picture into a binary image and analyzing particles bigger than 50 pixels.

### Chemical Analysis

In order to determine the Si concentration in the shoot and root, 200 mg dried and ground plant matter was digested in 3 ml 65% HNO_3_, 2 ml H_2_O and 2 ml 30% H_2_O_2_ in a microwave for 12 min at 190°C, then diluted with 20 ml 10% NaOH, neutralized with HNO_3_ (Haysom and Ostatek-Boczynski, 2006) and filled up to a final volume of 100 ml.

In order to determine the Fe, Cu, Mn and K, 50 mg of dried and ground shoot matter was digested in 2 ml 65% HNO_3_, 2 ml H_2_O, and 0.5 ml 30% H_2_O_2_ in a microwave for 25 min at 190°C and then diluted with distilled water to 25 ml.

The Si, Fe, Cu, Mn, and K in the plant extracts and nutrient solution were determined by ICP-MS (7500c Agilent Technologies, Santa Clara, CA, USA).

### Statistical Analysis

All treatments were replicated four times unless stated otherwise and the mean of the treatments were compared using *t*-test, Tukey or Bonferroni test using Sigma Plot (Systat Software Inc., San Jose, CA, USA). A cumulative link mixed model was calculated with *p* < 0.05 using the package ordinal in R Software (R Development Core Team, 2011) for comparison of the developmental stages of CB. The statistical qRT-PCR analysis was performed using the R-Macro of Steibel et al. (2009).

## Results

A total of ten homozygous rice lines were investigated to observe the function of Si enhanced genes involved in the lignin and suberin synthesis (**Table 2**). Only one KO (1B-14436) and one OE (3A-16329) line showed a significant reduction or increase of the transcript level compared to WT for the ABC transporter *OsABCG25* (LOC_Os10g30610).

**Table 2:** Homozygous rice insertion lines and target genes related to suberin and lignin synthesis, gene loci and changes in target gene transcription relative to wild type (WT) plants.

Insertion line	Target gene	Gene Locus	Transcription of target gene in insertion line relative to WT
2D-41110	Phenylalanine ammonia-lyase	LOC_Os02g41680	1.44 n.s.
3A-14487	4-coumarate-CoA ligase-like 6	LOC_Os01g67540	0.53 n.s.
3A-01911	Class III peroxidase	LOC_Os06g16350	1.83 n.s.
3A-08589	Class III peroxidase	LOC_Os08g02110	1.05 n.s.
1B-14436 (KO)	ABC transporter OsABCG25	LOC_Os10g30610	0.21^∗∗∗^
3A-06124	ABC transporter OsABCG25	LOC_Os10g30610	1.05 n.s.
3A-16329 (OE)	ABC transporter OSABCG25	LOC_Os10g30610	5.01^∗∗∗^
3A-02127	ABC transporter	LOC_Os10g30610	1.7 n.s.
3A-60593	ABC transporter	LOC_Os10g30610	1.36 n.s.
M0066685	Leucine-rich repeat receptor kinase	LOC_Os11g14050	1.78 n.s.

*n.s, not significant (*p* > 0.05); ^∗∗∗^significant with *p*-Value < 0.05.*

**FIGURE 1:**
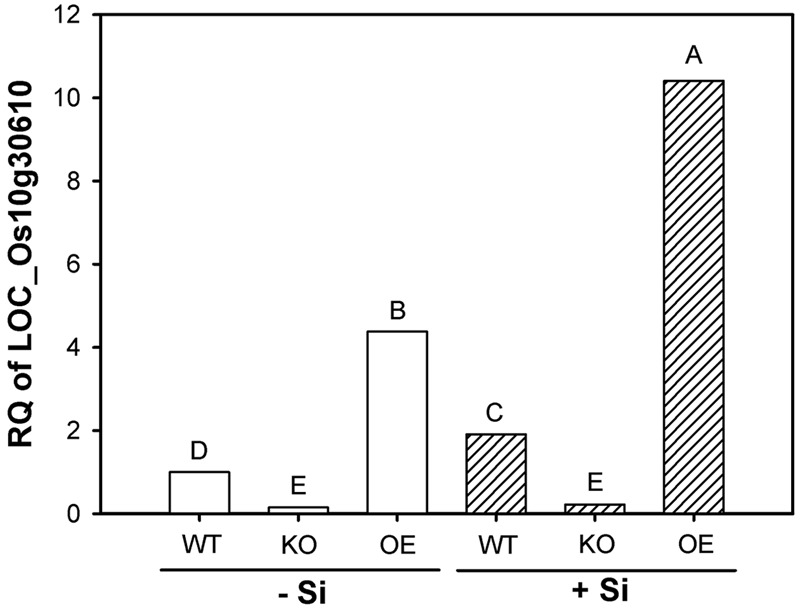
**Transcription of the gene LOC_Os10g30610 (OsABCG25) in root zone 4–6 cm of wild type (WT), knockout (KO) line 1B-14436 and overexpression (OE) line 3A-16329 (OE) plants grown in nutrient solution with low (–Si, 3 mg Si L^-1^) and high (+Si, 30 mg L^-1^) Si supply.** Relative quantity (RQ) of WT –Si = 1. Different letters indicate significant differences between treatments at *p* < 0.01.

Results given in **Figure 1** confirm down- and upregulation of this gene in the respective mutant at low and high Si supply. The expression in OE plants was increased by a factor of four at both Si levels, whereas the transcript level in KO plants was reduced to 1/6. High Si supply enhanced the gene expression by a factor of 2 in WT and OE plants, but did not in KO plants.

However, there was no pronounced effect of differential gene expression in mutants on suberin fractions in outer cell layers (OPR). The Total content of ω-hydroxy fatty acids and 2-hydroxy fatty acids, and fractions of C24 acids, C24 alcohols, C24 diacids, ω-hydroxy fatty acids and 2-hydroxy fatty acids were not significantly affected (Supplementary Figures S2, S3). The content of aromatic compounds, both coumaric acid and ferulic acid, in the OPR was decreased in the KO mutants, but the OE mutants did not differ clearly from the WT plants (Supplementary Figure S4).

**FIGURE 2:**
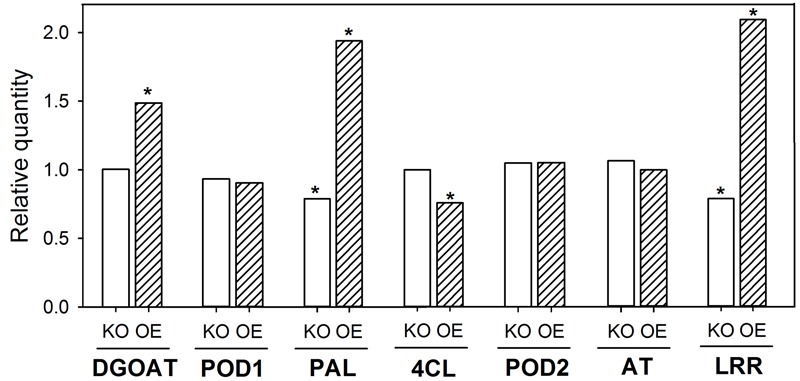
**Transcription of Casparian band (CB)-related genes in root zone 4–6 cm of WT, KO line *1B-14436* and OE line *3A-16329*.** The OE plants were grown in nutrient solution with low (–Si, 3 mg Si L^-1^) Si supply and KO grew in high (+Si, 30 mg L^-1^) Si supply. The RQ of corresponding WT (–Si /OE; +Si/KO) = 1. Abbreviations of the genes are: 4CL, 4-coumarate ligase (LOC_Os01g67540); AT, glycerol-3-phosphate acyltransferase (LOC_Os05g20100); DGOAT, diacylglycerol O-acyltransferase (LOC_Os06g22080); LRR, leucine-rich repeat family protein (LOC_Os11g14050); PAL, phenylalanine-ammonia-lyase (LOC_Os02g41680); POD1 and 2, peroxidase (LOC_Os08g02110 and LOC_Os06g16350). A ^∗^indicates significant differences between the WT and mutant plant at *p* < 0.01.

The downregulation of one gene involved in CB development should result in an altered regulation of other genes known to be involved in CB development from previous studies (Fleck et al., 2011). The KO of *OsABCG25* resulted in a downregulation of PAL (LOC_Os02g41680) and LRR (LOC_Os11g14050) by 30% (**Figure 2**). The OE resulted in an upregulation of DGOAT (LOC_Os06g22080), PAL and LRR by a factor of 1.5 to 2. Additionally, 4CL (LOC_Os01g67540) was slightly downregulated. Moreover, POD1 and 2 (LOC_Os08g02110 and LOC_Os06g16350) and AT (LOC_Os05g20100) were not regulated.

**FIGURE 3:**
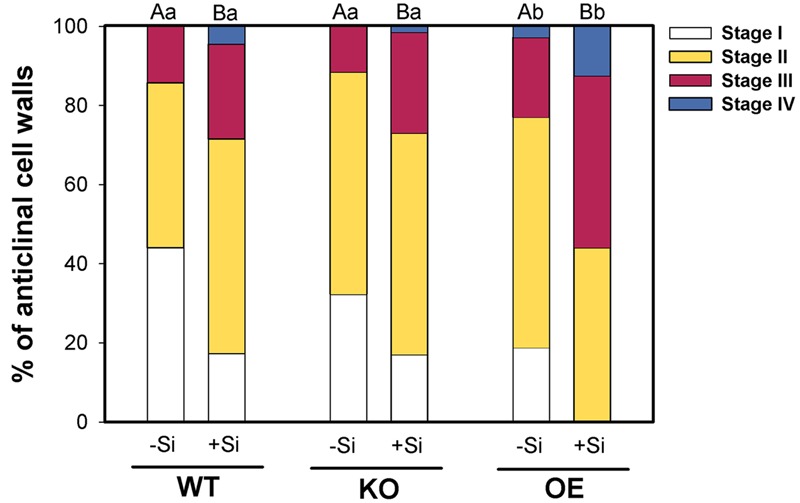
**Development of CB in the exodermis of WT plants and the mutant lines *1B-14436* (KO) and *3A-16329* (OE) as affected by Si supply (concentration are given in the legend of **Figure 1**).** The root zone investigated was 4–6 cm behind the root tip. The CB formation was classified in four stages: I:0, II: 0–25, III: 25–50, IV: >50% of the length of the anticlinal cell wall with CB *n* = 4. Different capital and small letters indicate significant differences between Si treatments within a genotype and for genotypes within the same Si level, respectively; cumulative link mixed models with *p* < 0.05.

Furthermore, microscopic evaluation of the exodermis development in berberin-aniline stained hand cuttings of the zone 4–6 cm behind the root tip showed different patterns of CB formation (**Figure 3**). High Si supply enhanced in WT and the mutants the CB development which was reflected in a decreased number of cell walls without CB and a higher number of cell walls of Stage III or even fully developed CB. Furthermore, the CB formation in the OE mutant compared to the WT was significantly enhanced in both Si levels. However, the KO mutant was not different from the WT.

**FIGURE 4:**
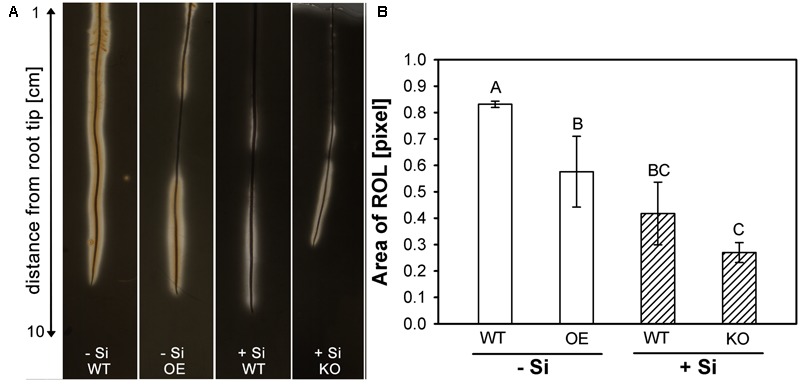
**(A)** Visualization of radial oxygen loss (ROL) from rice roots using a FeS-agar. The WT plants were grown with low (–Si) and high (+Si) Si supply (conc. are given in the legend of **Figure 1**), whereas the insertion line 1B-14436 (KO) was cultivated only in + Si and mutant 3A-16329 (OE) only in –Si; **(B)** Area of ROL of roots of WT plants and the mutation lines *1B-14436* (KO) and *3A-16329* (OE). Different letters indicate a significant difference between treatments; Tukey test with *p* < 0.05.

These findings agreed well with the observations on ROL from rice roots (**Figure 4**). Rice roots were embedded in iron-sulfur agar to visualize the ROL as a clear zone along the root. The KO line was investigated under +Si conditions, since a fully developed ROL can be already expected for WT roots under -Si conditions. By contrast, ROL was investigated for the OE line under -Si, since the ROL of WT roots under +Si is already heavily restricted to the 5 cm behind the root tip (Fleck et al., 2011).

The results confirmed previous reports (Fleck et al., 2011, 2015) that Si reduces the ROL from WT roots. The clear zone in -Si plants, extended along the whole root length and even precipitation of Fe^3+^ hydroxide/oxide was visible at the root surface. The area of ROL was reduced by half under high Si and restricted to the first 5 cm behind the root tip. The OE line had a clearly reduced ROL compared to WT, whereas the KO mutant behaved like the WT.

**FIGURE 5:**
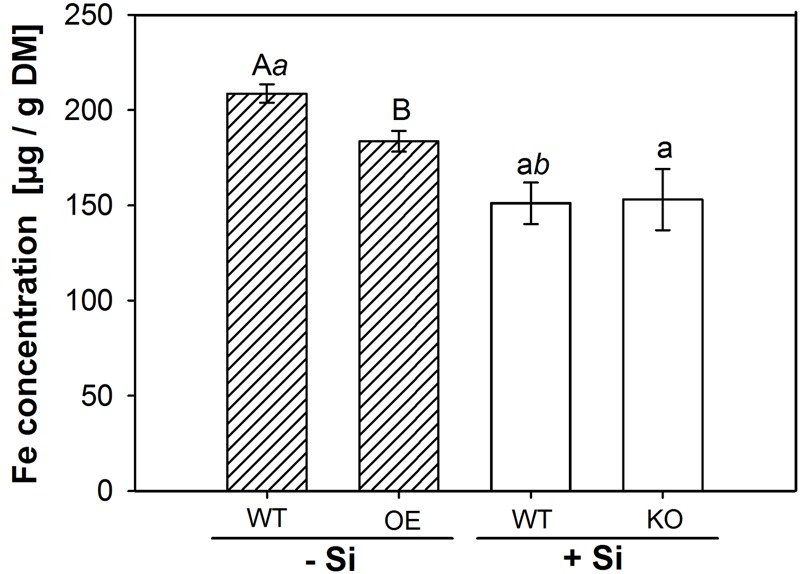
**Iron content in shoot dry matter.** The WT plants were grown with low (–Si) and high (+Si) Si supply (conc. are given in the legend of **Figure 1**), whereas the insertion line 1B-14436 (KO) was cultivated only in +Si and mutant 3A-16329 (OE) only in –Si. Different capital, small and italic letters indicate a significant difference between treatments; students *t*-test with *p* < 0.05.

Silicon application was reported to decrease the Fe concentration in rice shoots (Ma and Takahashi, 1990; Dufey et al., 2013). We speculated that this effect might be related to the CB-formation and analyzed the Fe concentration in shoot matter (**Figure 5**). Si application reduced Fe concentration in WT shoots by 20%. A significant reduction compared to the WT under -Si was also observed in the OE mutant, whereas the KO line was not different to the WT under +Si.

## Discussion

### Gene Expression

The KO line and one OE line out of nine homozygous lines showed a significant differential expression of the gene of interest indicating a very low efficiency of the CaMV 35s promoter enhancer-trap transgenic lines (**Table 2**). The CaMV 35s was described to be less active in monocots, roots and rhizodermal cells (Battraw and Hall, 1990; McElroy et al., 1990). Obviously, CaMV 35s enhancer-trap transgenic lines were not suitable to investigate gene function in rice roots. Additionally, we observed only heterozygous progenies for some CaMV 35s enhancer trap-lines (data not shown). The reasons could be lethality of homozygous plants and/or apomixis (Bicknell and Koltunow, 2004).

**FIGURE 6:**
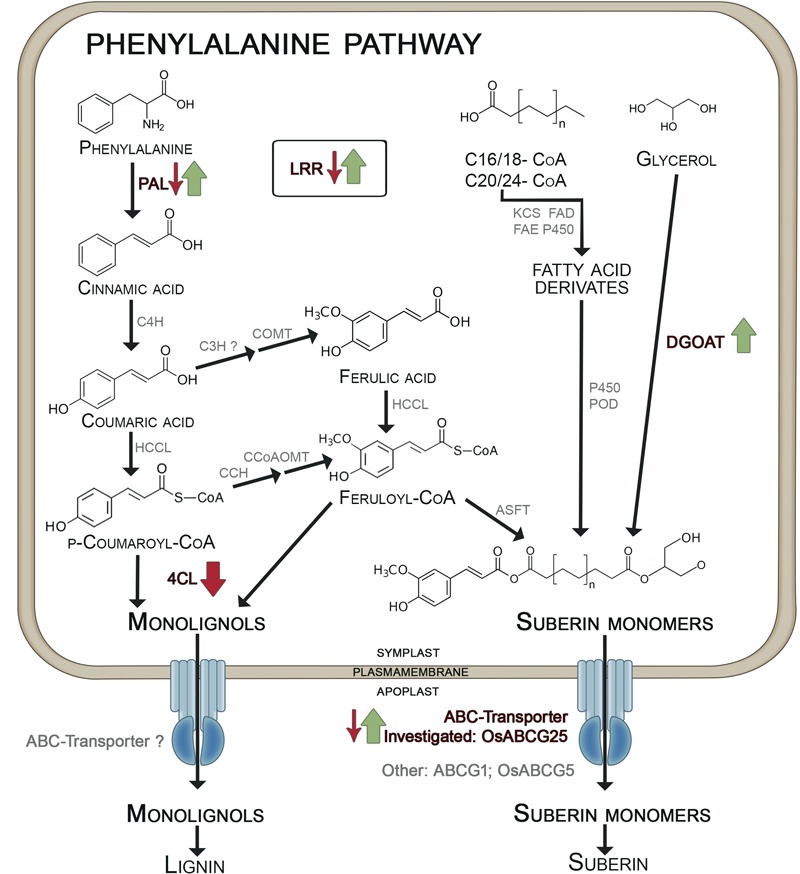
**Differential expression of genes involved in exodermal CB development in mutants of OsABCG25.** KO mutant: small arrows; OE mutant: big arrows. Phenylalanine-ammonia-lyase (PAL) catalyzes the formation of cinnamic acid which is metabolized by cinnamate-4-hydroxylase (C4H) to coumaric acid. Ferulic acid is built by coumaroyl-CoA-3-hydroxylase (C3H) and caffeic acid O-methyltransferase (COMT). Hydroxycinnamate-CoA-ligase (HCCL) catalyzes the formation of coumaryl-CoA and feruloyl-CoA which are converted to monolignols by 4-coumarate ligase (4CL) (Zhong et al., 1998; Eckardt, 2002). From coumaryl-CoA also feruloyl-CoA is generated by p-coumaroyl CoA 3-Hydroxylase (CCH) and caffeoyl-CoA O-methyltransferase (CcoAOMT) which is bound to suberin monomers by aliphatic suberin feruloyl transferase (ASFT). The aliphatic components of suberin monomers originate from fatty acid derivates and glycerol, which is incorporated by diacylglycerol O-acyltransferase (DGOT) (Franke et al., 2005; Franke and Schreiber, 2007; Li-Beisson et al., 2013). Both, monolignols and suberin monomers are transported by ABC-transporter from the symplast into the apoplast. We suggested that the investigated OsABCG25 transports suberin monomers. Other suberin transporters are ABCG1 (Landgraf et al., 2014) and OsABCG5 (Shiono et al., 2014). Further proteins are: CCH, p-coumaroyl CoA 3-hydroxylase; FAD, fatty acid desaturase; FAE, fatty acid elongase; KCS, β-ketoacyl-Coa synthase; LRR, leucine-rich repeat family protein; P450, cytochrome P450 monooxygenase; POD, peroxidase. Arrows indicate significant regulation in KO/OE mutants vs. +WT/-WT (*p*-value > 0.01), green stands for upregulation, red stands for downregulation. The scheme based on Eckardt (2002) and Franke and Schreiber (2007).

The KO and OE of the ABC transporter *OsABCG25* (LOC_Os10g30610) resulted in down- and upregulation of some genes involved in the lignin and suberin metabolism, as summarized in **Figure 6** (**Figures 1, 2**). The PAL (LOC_Os02g41680), catalyzing the first step of the phenylpropanoid pathway, and the LRR receptor-like kinase (LOC_Os11g14050) were downregulated in KO, but upregulated in OE. Furthermore, the 4CL (LOC_Os01g67540) in OE was downregulated and an upregulation of DGOAT (LOC_Os06g22080) was observed. The 4CL metabolizes p-coumaric acid and ferulic acid to monolignols (Gross and Zenk, 1974). DGOAT is most probably involved in the esterification of glycerol compounds into aliphatic suberin precursors in roots (Cases et al., 1998; Lu et al., 2003), to which ferulic acid is transferred by aliphatic suberin feruloyl transferase (Molina et al., 2009) to generate suberin monomers. These observations suggest that the formation of monolignols in the OE mutant was reduced, whereas the pathway to suberin monomers was enhanced, leading to the speculation that suberin monomers are the substrate of the OE ABC transporter *OsABCG25* (**Figure 6**). This transporter has no sequence similarity to *ABCG1*, a suberin transporter which has already been described from *Arabidopsis thaliana* (*ABCG1*, Landgraf et al., 2014). Furthermore, the ABC transporter *OsABCG5* (LOC_Os03g17350) shows 72% sequence similarity on a protein level to the ABC transporter in this study. *OsABCG5* is necessary for the CB formation in the exodermis (Shiono et al., 2014), confirming the need of the ABC-transporter for suberin- transport and subsequent CB development.

The LRRs are widely described to be involved in many functions, such as signal transduction, sensing, pathogen-response (Shiu and Bleecker, 2001; Shiu et al., 2004) or even development of root exodermal cells (Huang et al., 2012). It was shown that another LRR, the *Docs1*, belonging to the LRR RLK group in the LRR II subfamily, is necessary for the formation of sclerenchyma and exodermis in young lateral roots of rice. The LRR in this study belongs to the same structure group and subfamily with an LRR-domain, protein-kinase and transmembrane domain similar to *Docs1*. The LRR was upregulated through Si and by OE of the ABC-transporter. The LRR was downregulated in KO. This indicates a role of LRR in CB development.

### Suberization, Oxidation Power, and Fe Uptake

The suberin analysis in this work showed the typical pattern of rice root aliphatic components (Schreiber et al., 2005a,b). The OPR contained the typical abundant ω-hydroxy acids for rice in C16, 28 and 30. C24 diacids also appeared as expected, but were not different between genotypes (Supplementary Figures S2, S3). However, the total aromatic suberin amount, both coumaric acid and ferulic acid, was lower in KO (Supplementary Figure S4). This may indicate a general disturbance of cell wall metabolism in OPR.

The supply with Si enhanced the development of the CB (**Figure 3**), as was previously described. It was hypothesized that Si crosslinks phenols with cell walls or induces precipitation of the phenols leading to an enhanced formation of CB (Fleck et al., 2011, 2015). Furthermore, it was shown that Si promotes the deposition of aliphatic and aromatic compounds synchronously in the exodermis as investigated by serial cuttings stained with Berberine-Aniline-Blue and Fluorol Yellow 088 (Fleck et al., 2015). Contrarily, *Arabidopsis thaliana*, a species without CB as an exodermal diffusion barrier, first develops lignin monolayers and later suberin lamellae in the endodermal CB (Naseer et al., 2012). Thus, in the early stages of CB development, CB in the exodermis consists of both suberin and lignin, while it is lignin in the endodermis.

A significant enhanced development of the CB could also be observed in the OE mutant, but no difference compared to the WT occurred in the KO mutant (**Figure 3**). These findings agree well with the fact that the OE of the ABC transporter stimulated the expression of PAL and DGOAT, supplying metabolites for the formation of suberin (**Figure 2**). The CB development in the KO plant was not different from the WT, suggesting that the function of the knocked-out transporter may be substituted by other ABC transporters.

The different CB in Si-supplied WT and in mutants was also reflected in the area of the ROL (**Figure 4**). The enhanced CB development in Si-supplied WT plants and in the OE mutant resulted in a clearly decreased ROL. The WT plants grown in -Si solution had an oxidation zone along the whole root length, whereas the oxidation zone in WT/+Si and OE/-Si was limited to the first 5 cm from the root tip and to the zone 7–10 cm, where lateral root development starts. The oxidation zone was unaffected under +Si in the KO mutant; this fits with the observation that CB development was not affected. Oxygen-transport in rice is provided by aerenchyma from the upper plant organs to the root tip (Nishiuchi et al., 2012). Exodermal CB functions as a diffusion barrier reducing the ROL (Kotula et al., 2009; Fleck et al., 2011). Differential CB development depending on Si supply and mutant was also paralleled by variations of the Fe concentration in shoot matter, which was reduced in WT plants by Si supply and in the OE mutant, but was unaffected in the KO mutant (**Figure 5**). This agrees with previous findings that Si supply in the nutrient solution reduces the concentration of Fe, Mn and other nutrients by 20–50% in leaf DM (Ma and Takahashi, 1990; Dufey et al., 2013). Si, supplied as monosilic acid, can bind with Cu^2+^, Mn^2+^ or Fe^2+^ (Stevic et al., 2016). Such binding to monosilic acid may also occur with Fe^EDDHA^, as was supplied in this research, and may decrease the bioavailability in rice, a strategy II species. Iron uptake in strategy II species grown in nutrient solution is assumed to take place through binding of Fe^3+^ to deoxymugineic acid in the apoplast of roots (Takagi, 1976; Bienfait et al., 1985) and subsequent uptake by yellow stripe-like transporters (Koike et al., 2004; Inoue et al., 2009; Lee et al., 2009). The flux of Fe from the nutrient solution into the apoplast may be impaired by the Si-enhanced development of the exodermis. This conclusion is supported by the observation in the OE mutant that the increased development of exodermal CB resulted in a decreased Fe concentration in shoot matter (**Figure 6**), which is in line with the consideration of the exodermal CB as a diffusion barrier controlling the ion flow into the apoplast (Faiyue et al., 2010). Exodermal CB hampers the apoplastic pathway of water and Na uptake (Yeo et al., 1987; Steudle, 2000) and it was shown that Si supply reduces the apoplastic Na transport across the root of rice (Yeo et al., 1999; Gong et al., 2006; Krishnamurthy et al., 2009). The presented research confirms the function of OsABCG25 and indicates the involvement of genes related to the phenylpropanoid pathway, such as PAL, DGOAT and 4CL in the Si-promoted formation of CB. The OE of the ABC transporter as well as silicic acid supply enhanced the CB formation in the exodermis leading to a decrease of both, ROL and Fe uptake. This supports the view that the exodermis acts as diffusion barrier controlling fluxes in and out of roots.

## Author Contributions

MH, MS, and AF designed the research; MH, AF, EB, and NN performed the experiments; LS provided reagents and helpful discussions and MH, MS, and AF wrote the manuscript.

## Conflict of Interest Statement

The authors declare that the research was conducted in the absence of any commercial or financial relationships that could be construed as a potential conflict of interest.

## Footnotes

1 http://orygenesdb.cirad.fr/
