# Supplementary material for: An ABC Transporter Is Involved in the Silicon-Induced Formation of Casparian Bands in the Exodermis of Rice
Source: Front Plant Sci. 2017 Apr 28;8:671. doi: 10.3389/fpls.2017.00671 (PMC5408559; doi:10.3389/fpls.2017.00671)
Supplement: Supplementary file 1 [file Data_Sheet_1.DOCX]

Supplementary Material

An ABC Transporter is Involved in the Silicon-Induced Formation of Casparian Bands in the Exodermis of Rice

Martin Hinrichs^1∆*,^ Alexander T. Fleck^1∆^, Eline Biedermann^1^, Ngoc S. Ngo^1^, Lukas Schreiber^2,^ Manfred K. Schenk^1^

^∆^ These authors contributed equally to this work

^1^ Institute of Plant Nutrition, Faculty of Natural Science, Leibniz Universität Hannover, Hannover, Germany

^2^Institute of Cellular and Molecular Botany, Department of Ecophysiology, University of Bonn, Bonn, Germany

*** Correspondence:**Martin Hinrichs
hinrichs@pflern.uni-hannover.de

# Supplementary Data

**Supplementary Tab. 1:** Left and right primer of the wildtype primer pair (W-primer) for all mutant lines

| **Mutant line** | **Sequence of left primer** | **Sequence of right primer** |
| --- | --- | --- |
| *1B-04415* | ATGACGTGGTGGCTGACATA | GTCGGACACTCCGGAGATAA |
| *3A-14487* | TAGTGCTCCACTGGTTGCTG | CCCACTGATCTCTCTCTCGC |
| *2D-41110* | TGTGGTTCAATTAAACGGCC | AGGAGTACTCGCAATCCAGC |
| *M0060856* | GCGCCTACTGACCTACGTATG | CTCGCACTCCATTTCAGTACC |
| *4A-50856* | TCAAGAAACCCTCGACCATC | TCGTACCAGCTAGGACTCAAAG |
| *5A-00450* | TCAATTCGATCCGTTAATTCG | TGCCCTCAACAACACACAAT |
| *5A-00464* | AATTTAAGGGCGCCATGCTG | CACATTTGGTGGTGTTCGGA |
| *3A-01911* | CCCAAATGCAAGAAACGATC | CTTCGCTTTGAAGGTCTTGC |
| *M0038578* | GAGCCTTGAATTTGGCAATC | CAAATCCCTTGGGTTGTGTC |
| *3D-01082* | CACCGCGAGTTCATTTCTTT | TGAGGATGGGTTACCGAAAA |
| *3A-01215* | CGAATGTTCAGAGACGGTGA | TATAGATGCGTGTCGCTTGC |
| *3A-02897* | ACGTGGCATCATTTTGACTG | TCAATTCACTCAAACTGCGC |
| *3A-08589* | TGCTAGTCACCGTTCACCAC | TTTTATCCGCATTGTACGGG |
| *3A-06124* | ATTTTTGGCATTCCTGCAAC | ATCAATCTGAACAGCCCCTG |
| *3A-16329* | CAGAGGCCAAGGAGTTGAAC | TGATGCCTGCAAGTTTCATC |
| *3A-16331* | CTGGGATCCAATCCATTCAG | GAGTAAGTTGCCACTCCCAAAC |
| *3A-02127* | GGCTAACGCAGACAACTGTG | CTCCGCATGGAGAAGAAGAC |
| *3A-60593* | CTCACCGGGAAGACGAGTAA | CAATTTGCAGCAACTGGATG |
| *2D-00893* | AGCGAAAGAGGGAAGTTGGT | CGAAAGGGAAGGAGAAGGAG |
| *M0033740* | ATGCGCGACGCGAACGACTT | GCACTGCATGACGCCCTCGT |
| *M0058854* | AACCGGAATCGGATCCACCGAA | ACAGCTTCATGGTCCGGCTAGT |
| *2A-20141* | GGGTGCTAGATTGAAATATGCC | AAAGGAAGGGTGATGACGTG |
| *M0066685* | TCGCGTAAATACGCAACATC | GGGAGGAGAGTTGGGAACTC |
| *1B-14436* | GGACAGTGCTTCCTCTCAGC | GGCGTTGCTTAGTTACCCTG |

**Supplementary Tab. 2:** Insert specific primers (I-primer)

| **Suitable for mutants from** | **Sequence** |
| --- | --- |
| Postech | AACGCTGATCAATTCCAC |
| Postech | CGCGGCGTGCTTTGGAAATGT |
| Postech | ACGAAATGGCCTCCTTGCGGT |
| TRIM | CGAAAAGTTCGACAGCGTCT |
| TRIM | ACATTGTTGGAGCCGAAATC |
| TRIM | CGAAAACGGCAAGAAAAGCAGTC |
| TRIM | AACGGTTCAGGCACAGCACATCAA |

**Supplementary Tab. 3:** Primer for qRT-PCR

| Gene | Identifier | Forward Primer [5'-3'] | Reverse Primer [5'-3'] |
| --- | --- | --- | --- |
| 4-coumarate--CoA ligase-like 6 (4Cl) | LOC_Os01g67540 | tgaggcaaccgggtgcatacctta | aagccacacggcgcactttctt |
| Phenylalanine ammonia-lyase (PAL) | LOC_Os02g41680 | tcacaagctcaagcaccatc | ctcaccaagcttcttggcat |
| Ef1alpha | LOC_Os03g08010 | tcaagtttgctgagctggtg | aaaacgaccaagaggagggt |
| Glycerol-3-phosphate acyltransferase (AT) | LOC_Os05g20100 | tgctgaacaagcccatcactgc | tggcgcaatcaactccgatacc |
| Diacylglycerol O-acyltransferase (DGOAT) | LOC_Os06g22080 | acatgttcgccatgaaccaggca | tgccctgttgggatcgaagcac |
| Peroxidase precursor (POD) | LOC_Os06g16350 | cagcgccatggacagccaca | acggtgtcggccgtggagta |
| Peroxidase precursor (POD) | LOC_Os08g02110 | tcctgaattgcccgccttagctct | tcacaaagacgcggccacgaaa |
| ABC-transporter | LOC_Os10g30610 | atcatctaatgaggcacggc | tcattgtctggctgcagaac |
| Leucine-rich repeat family protein (LRR) | LOC_Os11g14050 | atcaggcaccataccaagccagc | tgggaggaatgccgccagtgaaa |

**Supplementary Figure 1:** Silicon concentrations in shoot and root of wildtype (WT) plants and the insertion lines *1B-14436* (KO) and *3A-16329* (OE) grown in nutrient solution with or without Si supply. Different letters indicate a significant difference between Si treatments of a genotype; t-test with p < 0.05.


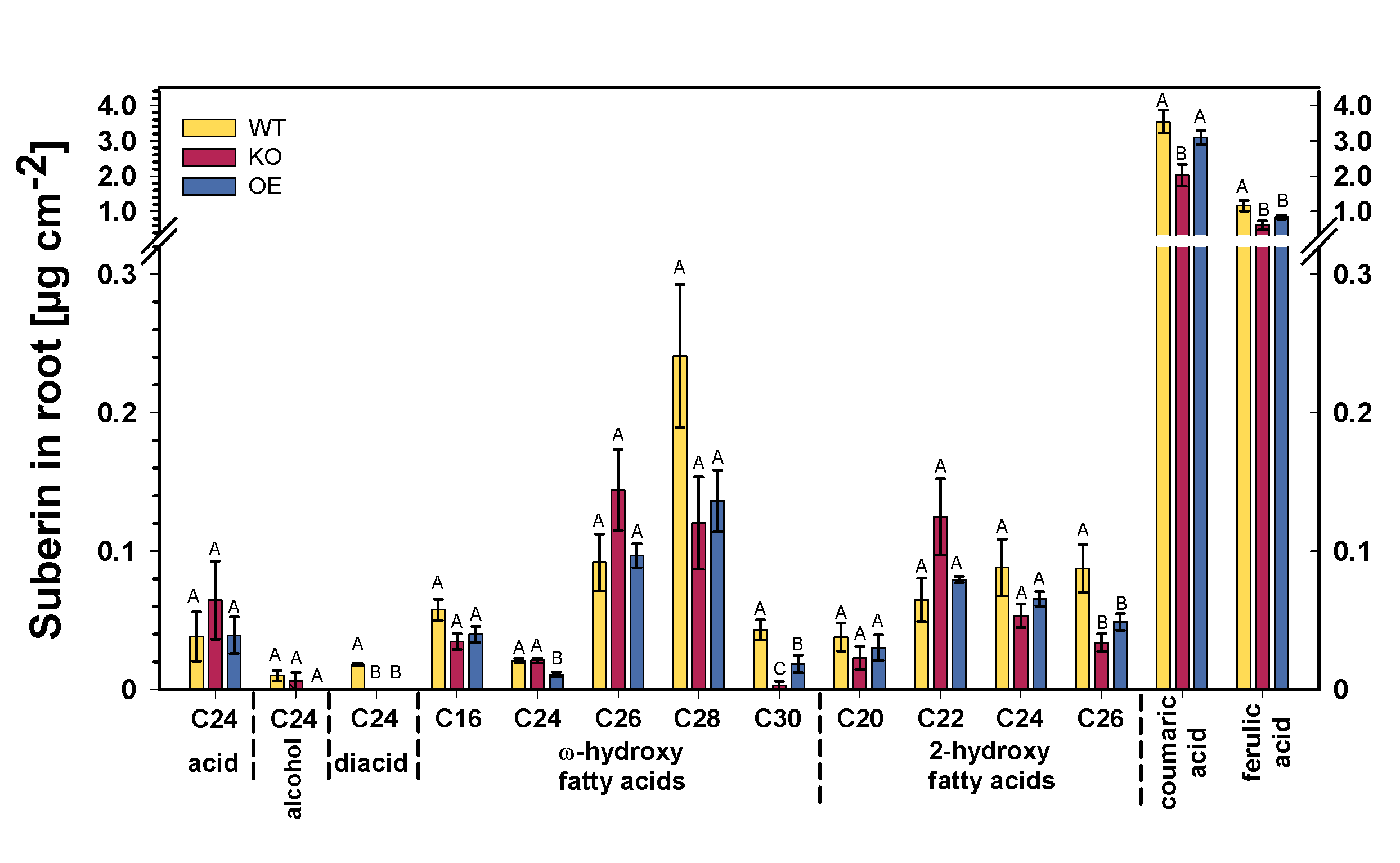


**Supplementary Figure 2:** Suberin amounts in the outer cell layers comprising the exodermis and sclerenchyma of root zone 4-6 cm in wildtype (WT) plants, knockout (KO) mutant, and overexpression (OE) mutant. Amounts were determined via GC-FID. Data are mean ± s.e., n = 4. Different letters indicate significant differences between treatments at p < 0.05 using Bonferroni-adjustment.


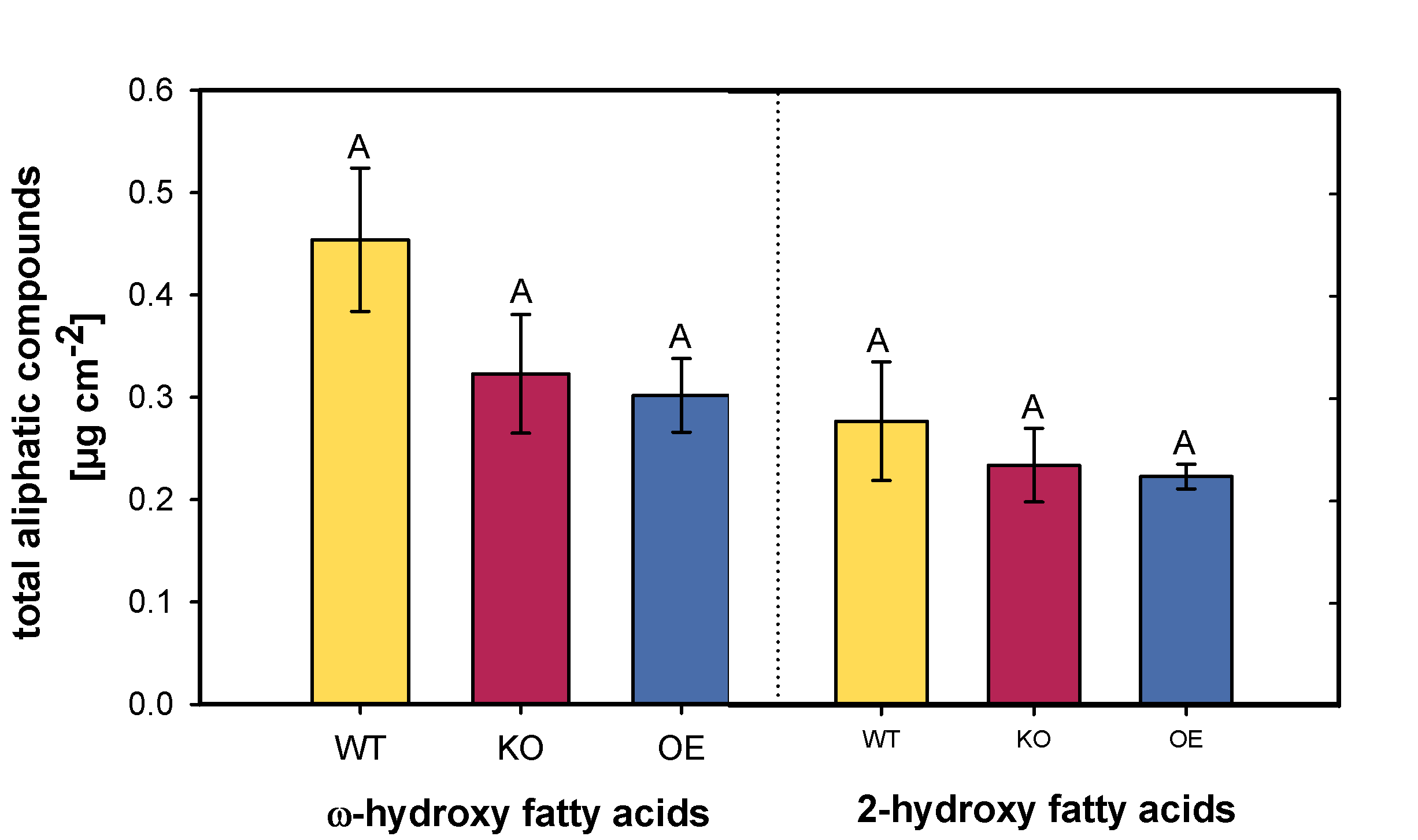


**Supplementary Figure 3:** Total aliphatic suberin amounts in the outer cell layers comprising the exodermis and sclerenchyma of root zone 4-6 cm in wildtype (WT) plants, knockout (KO) mutant, and overexpression (OE) mutant. Amounts were determined via GC-FID. Data are mean ± s.e., n = 4. Different letters indicate significant differences between treatments at p < 0.05 using Bonferroni-adjustment.


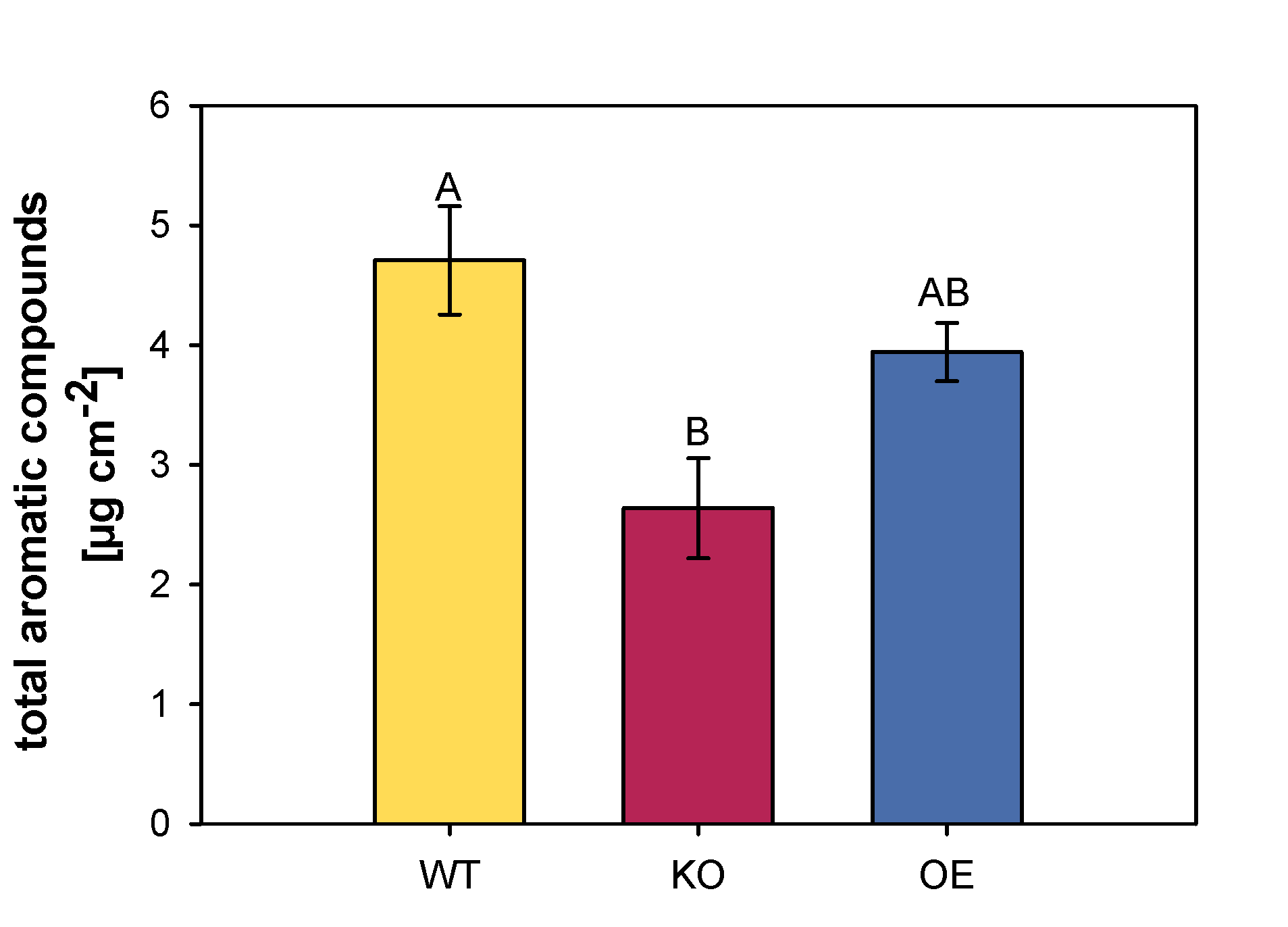


**Supplementary Figure 4:** Total aromatic suberin amounts in the outer cell layers comprising the exodermis of root zone 4-6 cm in wildtype (WT) plants, knockout (KO) mutant, and overexpression (OE) mutant. Amounts were determined via GC-FID. Data are mean ± s.e., n = 4. Different letters indicate significant differences between treatments at p < 0.05 using Bonferroni.
